# Comparative transcriptional analysis of *Bacillus subtilis* cells overproducing either secreted proteins, lipoproteins or membrane proteins

**DOI:** 10.1186/1475-2859-11-66

**Published:** 2012-05-24

**Authors:** Bogumiła C Marciniak, Hein Trip, Patricia J van-der Veek, Oscar P Kuipers

**Affiliations:** 1 Department of Molecular Genetics, Groningen Biomolecular Sciences and Biotechnology Institute, University of Groningen, Groningen, The Netherlands; 2 Kluyver Center for Genomics of Industrial Fermentation, Delft/Groningen, The Netherlands; 3 Present address: Molecular Microbiology, Groningen Biomolecular Sciences and Biotechnology Institute, University of Groningen, Nijenborgh 7, 9747 AG, Groningen, The Netherlands; 4 Institute for Life Science & Technology, Hanze University Groningen of Applied Sciences, Zernikeplein 7, 9747 AS, Groningen, The Netherlands

**Keywords:** Protein overproduction, Secretion stress, Production host, *sigW*, *ykrL*, LiaRS, Membrane, Cell wall

## Abstract

**Background:**

*Bacillus subtilis* is a favorable host for the production of industrially relevant proteins because of its capacity of secreting proteins into the medium to high levels, its GRAS (Generally Recognized As Safe) status, its genetic accessibility and its capacity to grow in large fermentations. However, production of heterologous proteins still faces limitations.

**Results:**

This study aimed at the identification of bottlenecks in secretory protein production by analyzing the response of *B. subtilis* at the transcriptome level to overproduction of eight secretory proteins of endogenous and heterologous origin and with different subcellular or extracellular destination: secreted proteins (NprE and XynA of *B. subtilis*, Usp45 of *Lactococcus lactis*, TEM-1 β-lactamase of *Escherichia coli*), membrane proteins (LmrA of *L. lactis* and XylP of *Lactobacillus pentosus*) and lipoproteins (MntA and YcdH of *B. subtilis*). Responses specific for proteins with a common localization as well as more general stress responses were observed. The latter include upregulation of genes encoding intracellular stress proteins (*groES*/*EL*, CtsR regulated genes). Specific responses include upregulation of the *liaIHGFSR* operon under Usp45 and TEM-1 β-lactamase overproduction; *cssRS*, *htrA* and *htrB* under all secreted proteins overproduction; *sigW* and SigW-regulated genes mainly under membrane proteins overproduction; and *ykrL* (encoding an HtpX homologue) specifically under membrane proteins overproduction.

**Conclusions:**

The results give better insights into *B. subtilis* responses to protein overproduction stress and provide potential targets for genetic engineering in order to further improve *B. subtilis* as a protein production host.

## Introduction

The Gram-positive bacterium *B. subtilis* is widely used in large scale production of endogenous and heterologous proteins used in food- and other industries. It is particularly favored as a production host since it has the capacity of secreting proteins to high levels into the medium enabling easy isolation and purification, it can be grown in large fermentations and is considered as a GRAS (Generally Recognized As Safe) organism by the US Food and Drug Administration. In addition, *B. subtilis* is still the most studied Gram-positive organism in fundamental research and is therefore a good model organism in the search for bottlenecks in protein overproduction. There are several cellular mechanisms that can hamper secretion of heterologous proteins on particular stages of the *B. subtilis* secretion pathway. At early stages of protein secretion, like synthesis of secretory pre-proteins, pre-protein interactions with cellular chaperones and binding to the translocase, the limitations may potentially result from, e.g., low transcription levels, inefficient translation, presence of intracellular proteases, deficiency in chaperones, poor targeting to the translocase, *etc.*[1]. The second stage of the protein secretion, *i.e.* translocation across the membrane *via* the Sec- or Tat- [2] translocase, may be confined by secretion machinery jamming [1]. At the late stages, which include removal of the signal peptide, release from the translocase, folding and passing the cell wall, deficiency in signal peptidases, foldases, chaperones and presence of extracellular proteases resulting in incorrect folding of proteins and protein’s instability may also set limits to the secretion efficiency [1,3]. The focus on identification and later manipulation of factors involved in protein secretion have led to the improvement of *B. subtilis* as a production host, for example by deletion of extracellular and/or intracellular proteases [4-6], use of strong or inducible promoters [7-9], overproduction of chaperones [10,11] or signal peptidases [12,13], modification of the cell wall [14,15], protein modification [16,17] and deletion of stress responsive systems [18].

Next to overproduction of proteins secreted into the medium, the overproduction of membrane proteins in *B. subtilis* is of a particular interest [19]. Membrane proteins are potential drug targets as they are exposed to and accessible from the extracytoplasmic environment, and therefore interesting for the pharmaceutical industry. Rational drug design, however, requires a three-dimensional structure, usually obtained from protein crystals, which can only be obtained when sufficient amounts of membrane protein of high quality are available [19].

In this work, a comparative transcriptomics approach was followed to study cellular responses to secretory proteins overproduction at the transcriptional level, in order to reveal so far unidentified possible production bottlenecks and thus potential targets for productive host engineering. Endogenous and heterologous proteins with different subcellular localization, *i.e.* secreted proteins, membrane proteins and lipoproteins were overproduced in *B. subtilis.* At least two proteins of each localization were chosen, in order to be able to discriminate between effects specific for one protein and effects common to one localization class. Transcriptomes were analyzed using DNA microarrays and subsequent use of appropriate bioinformatics tools. General responses as well as responses specific to proteins with a particular localization were identified.

## Results and discussion

### Transcriptome analysis of lipoprotein, membrane protein or secreted protein overproduction stress

*B. subtilis* remains a powerful host for the (industrial) production of secreted or membrane proteins but expression of heterologous proteins in particular has met limitations. These may occur at different levels of the production and secretion pathway. Here, the response of *B. subtilis* on the transcriptional level to overproduction of secretory proteins of endogenous or heterologous origin and with different subcellular localization, *i.e.* membrane proteins, lipoproteins and secreted proteins, was determined by transcriptome analysis.

**Figure 1:**
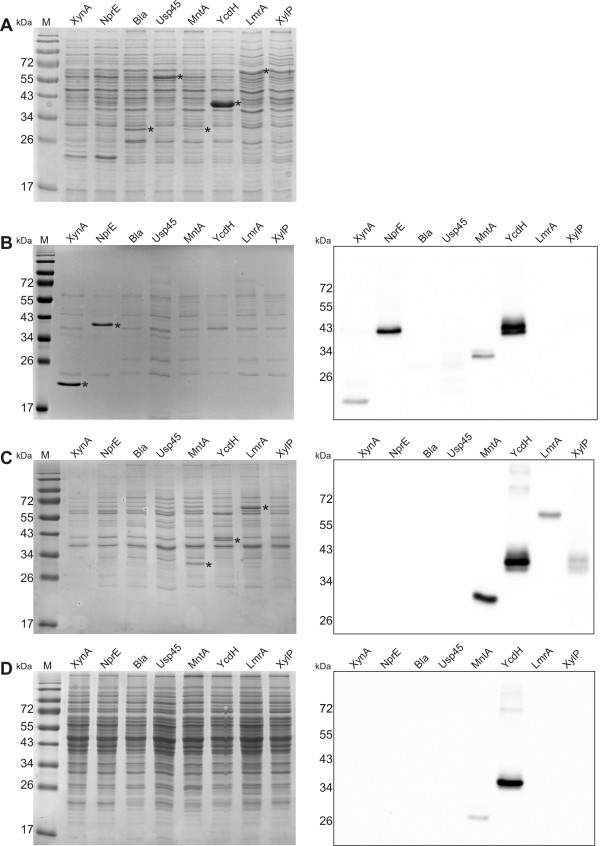
**Overproduction of different secretory proteins in *B. subtilis. ***(**a**) Whole cell extracts, (**b**) medium fractions, (**c**) membrane fractions, (**d**) cytoplasmic fractions. Left panels show SDS-PAGE gels; right panels (**b**, **c** and **d**) show immunodetection of the 6his-tagged proteins using Penta-His HRP conjugate antibodies (Qiagen). Asterisks indicate protein bands corresponding to the overproduced proteins. Calculated molecular masses of proteins, with and without signal peptide, in kDa: XynA, 32.3 (preprotein), 20.4 (matured); NprE, 56.5 (preprotein), 53.9 (matured); Bla (TEM-1 β-lactamase), 32.3 (preprotein), 29.7 (matured); Usp45, 48.2 (preprotein), 45.5 (matured); MntA, 33.4 (preprotein), 32.4 (matured); YcdH, 36.5 (preprotein), 34.3 (matured); LmrA, 66.2; XylP, 55.3.

Eight genes encoding heterologous and endogenous proteins (Table 1) with different subcellular localization were cloned using the SURE system overexpression vector pNZ8902 or pNZ8901 [7]: *lmrA* of *L. lactis*, encoding the membrane embedded putative multidrug transporter LmrA [20]; *xylP* of *Lb. pentosus* encoding a membrane embedded xyloside transporter XylP [21], *mntA* and *ycdH* of *B. subtilis* encoding the manganese binding lipoprotein MntA [22] and the putative zinc binding lipoprotein YcdH [23], respectively; *bla* of *E. coli* encoding the periplasm located TEM-1 β-lactamase (Bla) [24]; *usp45* of *L. lactis*, encoding the cell wall-associated Usp45 [25]; and *nprE* and *xynA* of *B. subtilis*, encoding the secreted neutral protease NprE [26] and the secreted xylanase XynA [27], respectively. The genes were fused to C-terminal 6His-tag encoding sequences. *B. subtilis* NZ8900 harboring these constructs or the empty vector pNZ8902 or pNZ8901 were grown to mid-exponential phase and expression was induced with subtilin. Samples were taken 30 min after induction for microarray analyses and after two hours for testing protein production. SDS-PAGE analysis of whole-cell, membrane, cytoplasm and medium fractions together with His-tag immunodetection demonstrated that XylP, LmrA, MntA, YcdH, TEM-1 β-lactamase and Usp45 were overproduced to levels varying from high for LmrA, YcdH and Usp45 to hardly visible on a Coomassie stained gel but well detectable using immunodetection (XylP) (Figure 1). Distinct localization patterns were observed for each class of protein (Figure 1). XynA and NprE were efficiently produced and secreted into the medium (Figure 1b), whereas Usp45 and TEM-1 β-lactamase were detected mainly in whole cell fractions (Figure 1a, left panel). Since the latter two were not or hardly detectable in the cytoplasmic and membrane fractions (Figure 1c and d), it is likely that they accumulated in the cell wall or membrane-cell wall interface. In accordance, TEM-1 β-lactamase expressed in *B. subtilis* was previously shown to accumulate in the membrane-cell wall interface due to inefficient passage through the cell wall [28]. Usp45 shows homology with proteins involved in cell wall metabolism, e.g., peptidoglycan hydrolases of *Streptococcus mutans*, *Streptococcus oralis*, *Lactococcus lactis subsp. lactis*[29-31], which may explain localization in or at the cell wall. Overexpression of *usp45* did not inhibit growth, whereas overexpression of *bla* resulted in growth inhibition as well as cell lysis, possibly due to interference with cell wall metabolism.

**Table 1:** **Proteins overproduced in *B. subtilis *NZ8900 host using SURE system**

**Protein**	**Function**	**Organism**	**Subcellular** **localization**
XylP	Xyloside transporter	*Lb. pentosus*	Membrane
LmrA (inactive mutant)	ABC-transporter	*L. lactis*	Membrane
MntA	Manganese binding	*B. subtilis*	Lipoprotein
YcdH	Zinc binding	*B. subtilis*	Lipoprotein
XynA	Xylanase	*B. subtilis*	Secreted
NprE	Neutral protease	*B. subtilis*	Secreted
Usp45	Unknown	*L. lactis*	Secreted
TEM-1 β-lactamase	β-lactamase	*E. coli*	Secreted

LmrA and XylP were exclusively found in the membrane fraction (Figure 1c, left and right panel). Similarly, the lipoproteins MntA and YcdH were present mainly in the membrane fraction, but immunodetection also indicated their presence at a low level in the medium (Figure 1b, right panel) and cytoplasmic fraction (Figure 1d, right panel). Immunodetection using His-tag antibodies proved to be of limited use in comparing levels of the different proteins, since they were detected with very different efficiencies (compare Figure 1b, left and right panel). Especially TEM-1 β-lactamase and Usp45 were hardly detectable in general.

The mRNA levels of each overproducing strain were compared with those of the control strain using DNA microarrays. Fold-changes in the expression level of genes that were at least 2.5 times up- or downregulated in response to overproduction of both proteins of the same subcellular localization, or to overproduction of at least 4 proteins with other destinations, are summarized in Table 2. Expression ratios of all the *B. subtilis* genes from eight microarray experiments are placed in Table S1 (Additional file 1). The complete microarray data is available at GEO repository (http://www.ncbi.nlm.nih.gov/geo/query/acc.cgi?acc=GSE34505) under accession number GSE34505.

**Table 2:** **Genes with significantly altered expression as a result of endogenous and heterologous proteins overproduction in *B. subtilis *cells**^***a,b***^

**Gene**	**Description**	**Overproduced proteins**							
		**Membrane proteins**		**Lipoproteins**		**Secreted proteins**			
		**XylP**	**LmrA**	**MntA**	**YcdH**	**XynA**	**NprE**	**Usp45**	**Bla**
**UPREGULATED**									
**Cell envelope stress response**									
*cssR*	two-component response regulator (class V)	0.96	1.06	**6.82**	1.38	**5.97**	1.08	**2.90**	1.85
*cssS*	two-component sensor histidine kinase (class V)	0.95	1.11	**3.10**	1.54	**2.67**	0.99	2.37	1.66
*htrB*	similar to HtrA-like serine protease (class V)	0.67	1.03	1.70	**7.37**	**10.86**	1.12	0.44	**7.52**
*htrA*	Quality control serine protease (heat-shock protein) (class V)	0.76	1.17	**3.17**	**5.18**	**14.88**	0.78	**46.70**	**7.23**
*sigM*	RNA polymerase ECF-type sigma factor	1.17	0.94	**3.01**	**2.51**	0.62	1.49	1.67	1.21
*liaI (yvqI)*	permease	2.38	1.07	1.31	**2.55**	1.59	2.03	**8.96**	**23.44**
*liaH (yvqH)*	modulator of liaIHGFSR (yvqIHGFEC) operon expression	2.32	1.08	1.19	**3.40**	1.47	**2.51**	**9.43**	**29.75**
*liaG (yvqG)*	hypothetical protein	1.48	1.06	2.27	**2.67**	0.82	**2.65**	**4.55**	**13.38**
*liaF (yvqF)*	integral inner membrane protein	0.89	1.07	2.14	1.94	0.92	**2.56**	**4.16**	**10.93**
*liaS (yvqE)*	two-component sensor histidine kinase [YvqC] sensing cell wall stress	1.14	NA	2.42	1.93	0.89	NA	**5.88**	**6.61**
*liaR (yvqC)*	two-component response regulator [YvqE] responding to cell wall stress	0.94	1.04	1.91	**2.57**	0.79	1.96	2.19	**4.76**
*ykrL*	Homolog of HtpX, membrane protease	**10.35**	**3.62**	1.66	0.88	2.15	0.90	**2.98**	1.85
									
*sigW*	RNA polymerase ECF-type sigma factor	**4.52**	**5.50**	1.95	1.77	1.62	1.05	1.73	2.44
*rsiW (ybbM)*	sigmaW anti-sigma factor	**5.54**	**4.11**	2.43	1.72	2.31	0.84	1.89	**2.61**
*yuaF*	putative membrane integrity integral inner membrane protein	**6.93**	**2.65**	1.09	0.23	1.41	1.31	2.15	2.36
*yuaG*	similar to flotillin 1	**12.21**	**3.43**	0.66	0.75	1.87	1.00	**4.52**	2.40
*yuaI*	putative acetyl-transferase	**15.16**	**6.62**	0.40	0.81	**2.80**	0.78	**5.01**	**2.70**
*ydjG*	putative phage replication protein	**7.70**	**2.85**	0.59	1.85	1.93	1.01	**3.09**	1.46
*ydjH*	hypothetical protein	**6.88**	**3.92**	0.60	0.48	1.68	1.09	2.19	1.86
*ydjI*	hypothetical protein	**6.78**	**4.55**	0.59	0.75	1.86	0.57	**2.84**	2.40
*yeaA*	conserved hypothetical protein	**5.58**	**3.28**	0.93	0.42	1.41	2.36	1.35	2.10
*ydjP*	similar to chloroperoxydase	**7.41**	**4.67**	1.16	0.91	**2.55**	1.53	**4.56**	**3.00**
*ydjO*	unknown	**3.81**	**7.59**	1.14	1.08	1.99	1.46	**2.97**	**2.57**
*sppA*	signal peptide peptidase	**4.25**	**3.12**	0.33	0.26	1.18	0.39	1.70	1.80
*yteJ*	integral inner membrane protein	**4.48**	**4.16**	0.31	0.29	1.27	0.34	2.27	1.77
*pbpE*	penicillin-binding protein 4	**11.25**	**8.56**	0.95	0.97	2.08	0.49	**6.37**	**2.72**
*racX*	amino acid racemase	**8.99**	**8.09**	0.78	0.95	2.06	0.45	**6.00**	2.39
*yaaN*	hypothetical protein	**4.03**	**6.82**	1.07	0.43	1.15	**2.64**	1.51	1.82
*yceC*	putative stress adaptation protein	2.44	**3.05**	2.06	NA	NA	0.54	NA	1.64
*yceD*	putative stress adaptation protein	**5.29**	**7.02**	0.81	0.76	2.04	0.92	**3.14**	1.55
*yceE*	putative stress adaptation protein	**4.55**	**5.36**	0.82	0.67	2.08	0.89	**3.71**	2.34
*yceF*	putative stress adaptation protein	**5.30**	**4.91**	0.70	0.78	2.07	0.73	**4.27**	1.79
*yceG*	hypothetical protein	**3.99**	**3.02**	0.79	1.07	1.94	1.04	**3.29**	1.22
*yceH*	hypothetical protein	**3.72**	**4.09**	0.64	0.99	1.99	0.60	**3.68**	2.15
*yjoB*	ATPase possibly involved in protein degradation	**7.16**	**2.98**	1.28	0.65	1.45	0.94	1.74	2.44
*yknW*	permease	**2.51**	**3.06**	0.67	0.36	1.12	0.84	1.14	1.46
*yknY*	ABC transporter ATP-binding protein	**2.63**	**2.54**	0.49	0.33	1.31	0.57	1.51	1.64
*ythQ*	putative ABC transporter (permease)	**10.01**	**5.75**	0.80	NA	2.21	NA	**3.21**	**3.19**
*yqfB*	hypothetical protein	**6.37**	**6.24**	1.12	0.56	1.66	0.39	**3.10**	**2.93**
*yvlA*	hypothetical protein	**4.11**	**3.06**	1.49	0.59	1.23	1.34	1.39	2.04
*yvlB*	hypothetical protein	**6.13**	**9.56**	0.99	0.66	1.76	0.91	**3.84**	2.10
*yvlC*	putative regulator (stress mediated)	**3.83**	**7.71**	0.99	0.61	1.61	0.76	2.24	2.05
*yvlD*	putative integral inner membrane protein	**4.26**	**6.66**	1.60	0.65	1.22	0.74	**2.98**	1.87
*yxjI*	unknown	**10.12**	**5.20**	0.96	0.55	1.60	1.46	1.38	1.83
									
*hrcA*	transcriptional repressor of class I heat-shock genes	**11.08**	2.23	1.89	**12.62**	0.96	1.30	2.33	2.12
*groES*	class I heat-shock protein (chaperonin)	**8.37**	**7.17**	0.10	**6.85**	**3.15**	1.11	**3.68**	**3.07**
*groEL*	class I heat-shock protein (chaperonin)	**8.38**	**4.17**	0.35	**5.07**	**2.73**	1.14	**3.00**	**2.66**
*nfrA*	FMN-containing NADPH-linked nitro/flavin reductase (class VI)	**7.36**	**3.32**	0.47	1.67	**10.37**	0.98	**13.77**	1.85
*ywcH*	putative monooxygenase (class VI)	**10.29**	**2.91**	1.04	**6.03**	**5.29**	0.86	NA	2.46
*trxB*	thioredoxin reductase	**4.71**	1.59	0.88	**2.98**	**3.97**	0.72	**3.56**	1.36
*trxA*	thioredoxin	2.29	1.94	0.86	1.68	**3.08**	1.13	**3.11**	1.31
*ctsR*	transcriptional regulator of class III stress genes	0.71	0.55	1.39	NA	NA	NA	NA	NA
*mcsA*	modulator of CtsR repression	**10.38**	**3.47**	0.87	**5.12**	1.72	1.57	**4.63**	**3.11**
*mcsB*	modulator of CtsR repression	**6.92**	**4.11**	0.83	**5.09**	2.46	1.09	**2.68**	**2.63**
*clpC*	class III stress response-related ATPase	**5.65**	**3.24**	0.65	**4.63**	2.03	0.80	**3.28**	**2.80**
*radA*	DNA repair protein	**2.79**	1.97	0.75	**3.86**	1.82	1.26	**3.14**	2.19
*clpE*	Class III, ATP-dependent Clp protease-like	**74.08**	1.89	0.98	**4.54**	0.77	1.24	**5.95**	**4.79**
									
*fdhD*	required for formate dehydrogenase activity	**2.89**	1.26	**2.99**	**4.65**	1.93	1.65	1.67	0.94
*cydB*	cytochrome bd ubiquinol oxidase (subunit II)	0.75	2.28	1.39	0.51	**2.93**	**4.50**	0.74	0.64
									
*kinD*	TCS sensor histidine kinase; initiation of sporulation	1.35	1.20	**3.18**	**2.76**	1.49	1.81	1.05	1.05
*yabT*	putative serine/threonine-protein kinase	2.19	1.14	NA	1.56	**2.55**	**2.65**	1.26	1.04
*yceK*	putative transcriptional regulator (ArsR family)	**2.58**	**2.54**	0.81	1.35	1.07	1.06	2.10	1.92
*yjbI*	putative thiol management oxidoreductase component	**3.93**	0.99	1.45	**2.95**	**3.23**	1.33	**3.61**	1.46
*yloC*	unknown	1.65	1.09	**3.56**	**3.51**	1.47	1.36	1.57	1.05
*yndN*	fosfomycin resistance protein FosB	**6.66**	**13.17**	0.94	0.32	1.60	0.56	**3.49**	**3.04**
*yrkA*	putative membrane associated protein	**3.20**	**3.75**	1.51	0.96	1.48	0.97	1.13	1.30
*yvdT*	uncharacterized transcriptional regulator (TetR/AcrR family)	**2.94**	**3.28**	1.82	2.19	1.37	1.80	2.26	1.92
*yvdS*	similar to molecular chaperone, putative membrane protein	**7.12**	2.12	1.20	1.14	1.08	1.26	**3.43**	1.96
*yvdR*	similar to molecular chaperone, putative membrane protein	**6.32**	**2.66**	2.46	1.39	1.01	1.36	**3.99**	1.81
									
*cydC*	ABC membrane transporter ATP-binding protein	**3.22**	**3.76**	0.87	0.32	**2.92**	2.36	2.25	1.12
*yhaT*	K+/H + antiporter for K + efflux	**15.45**	**3.48**	2.16	**2.91**	2.47	1.22	**4.80**	**2.54**
*yhaU*	K+/H + antiporter for K + efflux	**15.49**	2.19	2.38	1.84	**2.56**	1.00	**5.14**	1.97
**Unknown**									
*ydiN*	unknown	**4.44**	**2.76**	0.22	**5.11**	1.75	1.75	2.41	**3.51**
*yomP*	hypothetical protein	**3.57**	**3.21**	0.92	**3.33**	0.89	**2.60**	1.40	0.82
*yomT*	hypothetical protein	**2.92**	**2.60**	0.83	**2.90**	0.72	2.47	1.24	0.85
*yceI*	putative transporter	**2.92**	**3.55**	1.49	0.80	1.75	0.69	**2.89**	2.26
*yhaS*	K+/H + antiporter for K + efflux	**9.48**	2.13	2.08	1.76	2.42	0.96	**3.41**	1.94
*yomV*	hypothetical protein	**3.59**	**3.37**	1.05	**3.92**	0.73	**2.55**	1.43	0.88
*yomW*	hypothetical protein	**3.19**	**3.38**	0.97	**4.57**	0.65	**2.79**	1.31	0.78
*yomY*	hypothetical protein	**3.66**	**2.53**	1.09	**3.64**	0.77	**2.60**	1.66	0.82
*yonB*	hypothetical protein	**3.22**	**2.92**	0.96	**2.61**	1.01	2.26	1.24	0.79
*yonC*	hypothetical protein	**2.92**	**2.64**	0.83	**2.84**	0.96	2.41	1.43	0.84
*yvkN*	hypothetical protein	**3.21**	**2.69**	0.89	0.61	1.83	1.07	1.88	1.69
*ywmB*	hypothetical protein	1.32	1.18	**3.00**	**2.67**	1.30	2.43	0.55	1.16
									
*sdpA (yvaW)*	export of killing factor SdpC	**15.28**	**2.78**	**4.29**	**3.96**	**3.54**	1.54	**14.19**	**4.35**
*sdpB (yvaX)*	exporter of killing factor SdpC	**19.87**	**4.06**	**3.26**	**5.34**	**3.96**	1.49	**13.85**	**4.00**
*sdpC (yvaY)*	killing factor SdpC	**8.12**	**2.75**	2.45	**12.90**	**11.51**	1.21	**26.62**	**5.56**
									
*sppA*	signal peptide peptidase	0.24	0.32	**3.03**	**3.89**	0.85	**2.56**	0.59	0.56
*yteJ*	putative integral inner membrane protein	0.22	0.24	**3.18**	**3.51**	0.79	**2.93**	0.44	0.56
									
*gltT*	proton/sodium-glutamate symport protein	**3.36**	**3.12**	**2.52**	1.63	0.82	1.96	**8.35**	1.20
*pbuX*	xanthine permease	**3.32**	**2.74**	0.45	1.76	1.47	1.50	**4.45**	1.61
*yhaQ*	Na + −effluxABCtransporterATP-bindingprotein	2.07	1.55	**3.73**	**4.51**	2.19	2.10	1.65	1.32
*yoaG*	putative permease	0.13	0.67	**3.13**	**3.68**	1.20	1.15	0.66	0.53
									
*ctaB*	cytochrome caa3 oxydase assembly factor	1.18	1.63	0.66	1.16	1.34	1.15	**3.48**	1.17
*ctaC*	cytochrome caa3 oxidase subunit II	**4.37**	1.13	**4.74**	**2.68**	1.08	1.91	**5.52**	1.20
*ctaD*	cytochrome caa3 oxidase subunit I	**3.71**	1.37	**3.58**	**3.14**	NA	1.81	**3.33**	1.27
*ctaE*	cytochrome caa3 oxidase subunit III	**3.57**	1.44	2.40	**3.02**	1.90	2.08	**4.21**	1.10
*ctaF*	cytochrome caa3 oxidase subunit IV	**4.33**	1.91	2.05	**3.86**	**2.68**	2.32	**4.24**	1.41
*ctaG*	cytochrome aa3 assembly factor	**4.61**	NA	2.40	1.83	NA	1.19	**2.67**	NA
									
*bdbB*	bacteriophage SPbeta thiol-disulfide oxidoreductase	**3.59**	**2.79**	**6.00**	**2.57**	**3.05**	1.16	**14.00**	2.00
*wapA*	cell wall-associated protein precursor	**2.88**	1.07	1.15	**8.19**	**2.50**	0.91	**6.38**	1.15
*yxxG*	hypothetical protein	**4.04**	0.99	1.22	**8.68**	**4.20**	0.87	**5.25**	1.08
*wprA*	cell wall-associated protease	2.45	1.05	**4.87**	**4.19**	2.27	1.85	**9.13**	1.27
**Unknown**									
*yisL*	hypothetical protein	2.00	1.13	**2.58**	**4.59**	1.41	1.96	0.73	1.11
*yokE*	hypothetical protein	1.56	1.76	**7.19**	**2.84**	**2.74**	1.50	**4.35**	1.28
*ytxG*	hypothetical protein	1.80	0.76	**2.98**	**2.56**	0.83	1.00	0.48	0.93
*yukE*	hypothetical protein	1.37	1.56	**3.25**	**2.67**	0.92	2.04	1.60	1.02
*yxbC*	hypothetical protein	**3.55**	0.69	**3.22**	**5.71**	0.72	1.30	2.06	1.11

^*a*^ Significant changes (p value < 0.01, fold > 2.5) are shown in bold.

^*b*^ Endogenous proteins: MntA, YcdH, XynA, NprE; heterologous proteins: XylP (*Lb. pentosus*), LmrA and Usp45 (*L. lactis*), Bla (TEM-1 β-lactamase, *E. coli*).

### General effects

Overproduction of all secreted proteins, except NprE, caused upregulation of class I heat-shock genes coding for molecular chaperons *groES* and *groEL* (Table 2). Overproduction of the same proteins, except for XynA and MntA, resulted in activation of class III heat-shock genes, which code for components of protease complexes (ClpXP, ClpEP, *etc.*) [32,33] (Table 2), and other genes regulated by CtsR, a stress and heat-shock response regulator [32]. This intracellular stress response may be caused by a high protein production rate in combination with a limited capacity in protein secretion or membrane insertion, and/or, in case of the heterologous proteins, a lower compatibility of the secretion signal with the host secretion machinery. However, accumulation of the proteins was not observed (Figure 1d). This suggests that, although the proteins were apparently secreted with good efficiency, their presence at lower levels were enough to induce the general cytoplasmic stress response. Increased expression of chaperones like GroES/EL and Clp proteases can protect the cell from toxic accumulation of mis- or unfolded protein [34,35]. However, high expression and activity of proteases may also set a limit for production of heterologous proteins in *B. subtilis* on large scale.

The *nfrA-ywcH* operon, encoding a nitro/flavin reductase and a monooxygenase, respectively [36], was upregulated in 5 of the 8 cases (Table 2). NfrA is believed to be involved in a response to stress-induced protein damage and its corresponding gene is induced upon a wide range of stresses [37]. Therefore the coproduction of NfrA can be considered in the improvement of protein overproduction.

Another observed effect in case of most overproduced proteins was strong induction of the *yhaSTU* operon. It codes for a K^+^ efflux system and has been shown to be induced by alkaline pH, which has been suggested to be a secondary effect of compromised membrane function and bioenergetic integrity of the cell [38,39], and salt stress [40].

The genes *trxA* and *trxB* were upregulated in the majority of the cases, without a bias towards a particular localization of the overproduced protein. *trxA* and *trxB* are members of Spx regulon involved in thiol-specific oxidative stress and they code for thioredoxin and thioredoxin reductase, respectively [41]. These genes are thought to be required for keeping proteins in a reduced state which, once secreted, form disulfide bonds during folding [41]. However, there was no correlation between the presence of (putative) disulfide bonds in an overproduced protein and induction of *trxA* or *trxB* (only TEM-1 β-lactamase, YcdH and XylP possess putative disulfide bonds, out of which overproduction of only YcdH resulted in *trxB* induction). Therefore, upregulation of *trxA* and *trxB* is most likely induced by thiol stress as a result of secondary effects of overproduction of secretory proteins, such as a compromised membrane function.

An effect that was observed in case of all overexpressed proteins was strong downregulation of the *sdpABC* operon (sporulating delay protein operon) involved in production and secretion of the killing factor SdpC (Table 2). It plays a role in programmed cell death (PCD), a mechanism of sporulation delay by killing nonsporulating siblings and feeding on the dead cells under conditions of nutrient limitation [42,43]. This effect may be related to nutrient limitation which was shown to induce the sporulation process in a subpopulation of a *B. subtilis* culture with concomitant activation of the *sdpABC* and *sdpRI* immunity operons [43].

Another general effect, but less pronounced than for *sdpABC*, was downregulation of the *ctaCDEF* genes coding for cytochrome *c* oxidase *caa3*[44].

Overproduction of none of the proteins caused upregulation of genes coding for components of the secretion (Sec) machinery, like *secA*, *secDF*, *ffh, etc.*, which are responsible for translocation of unfolded pre-proteins across or insertion into the membrane (for review see [2]). Apparently, increasing its protein secretion capacity is not a strategy of the cell to deal with an accumulation of secretory proteins. This may indicate either that the SecYEG channel does not form a bottleneck in secretion in the experiments performed here, or that expression of the genes encoding the SecYEG components is simply not upregulated by (the consequences of) an artificially imposed overproduction of secretory proteins. The latter suggests that SecYEG should not necessarily be excluded as a potential target for production strain improvement. In agreement, overexpression of *prsA*, encoding the extracellular foldase PrsA, was shown to increase the secretion of an α-amylase fourfold [10], while *prsA* was not upregulated in any of the tested cases here. This however does not detract from the value of the data as a source of new potential targets for strain improvement. For some of these genes, induced by overexpression of many of the tested secretory proteins, it was indeed shown previously that either their deletion or overexpression improved specific protein production yields, e.g., *sigW* and *cssRS*[18] and genes encoding intracellular chaperones [5].

### Proteins with extracytosolic destination induce the CssRS mediated secretion stress response

Overproduction of the secreted protein XynA of *B. subtilis*, the cell wall-associated proteins Usp45 of *L. lactis* and TEM-1 β-lactamase of *E. coli*, as well as lipoproteins MntA and YcdH of *B. subtilis* resulted in significant upregulation of the secretion stress genes *htrA*, *htrB* and *cssRS* (Table 2). CssR and CssS encode a response regulator and its cognate, membrane embedded sensor, respectively, and control the expression of *htrA* and *htrB*[45,46]. These encode membrane-anchored HtrA and HtrB proteins, which have their active site on the *trans* side of the membrane and are thought to have proteolytic as well as chaperone activity for removal of misfolded protein or for assisting in folding of newly secreted proteins, respectively [47]. The CssRS two component system is activated by accumulation of mis- or unfolded secreted protein at the membrane - cell wall interface, as a result of, e.g., overexpression of these proteins or heat stress [48,49]. In this study, overproduction of the membrane proteins LmrA and XylP did not significantly induce *htrA* or *htrB*. This is in agreement with previous results from an analysis of the activation of the *htrA* promoter in response to overproduction of secretory proteins, including MntA, XynA, TEM-1 β-lactamase, Usp45 and LmrA, showing that the stress signal is sensed on the outside of the cell and not from within the membrane [48]. Surprisingly, NprE overproduction did not induce the CssRS response. Possibly, NprE can be produced and secreted to high levels without accumulation of misfolded protein.

### Usp45 and TEM-1 ÎÂ²-lactamase specifically induce the LiaRS-dependent response

The two proteins which were detected mainly in the whole cell fractions, but not in the membrane and cytoplasmic fractions, Usp45 and TEM-1-β-lactamase (Figure 1), specifically induced the *liaIHGFSR* (*yvqIHGFEC*) operon (Table 2), a cell envelope stress operon which is under control of the LiaRS (YvqCE) two-component system [50-53]. The fact that LiaRS is strongly induced by cell wall-active antibiotics [54], suggests that Usp45 and TEM1-β-lactamase had accumulated in or at the cell wall, as noted earlier, and thereby interfered with cell wall metabolism. Since the other secretory proteins did not, or to a much lesser extent, induce LiaRS (Table 2), it appears that the signal which is sensed by the sensor LiaS originates from cell wall metabolism related processes, rather than for example cell membrane integrity.

### Membrane protein overproduction induces a SigW response and *ykrL* expression

The overproduction of the membrane proteins LmrA and XylP and to a lesser extent the cell wall-associated proteins Usp45 and TEM-1 β-lactamase caused significant upregulation of *sigW* and many genes belonging to the SigW regulon (Table 2). The SigW regulon has been shown to be induced by a variety of cell envelope stresses like treatment with detergents (Triton X-100), antibiotics (vancomycin, penicillin) [51], alkaline stress [55] or membrane protein overproduction [18]. Activation of SigW depends on proteolytic degradation of the anti-SigmaW factor RsiW by a multipass membrane protease, PrsW and, subsequently, other proteases [56,57], but the exact signal triggering this cascade is not known. The induction by membrane protein overexpression suggests that the stress signal is sensed from within the membrane.

Next to the SigW response, an unknown gene, *ykrL,* was significantly upregulated under LmrA and XylP overproduction (Table 2). YkrL shows high homology to the *E. coli* HtpX, a membrane embedded metalloprotease, which has been implied in membrane protein quality control [58]. The upregulation of *ykrL* suggests a similar role in *B. subtilis*. It would be of interest to test the effect of different levels of YkrL on the level and quality of overproduced membrane proteins. Expression of *htpX* in *E. coli* is regulated by the CpxRA two component system that regulates a number of genes involved in cell envelope stress, including *degP* (or *htrA*), encoding a close homologue of *B. subtilis* HtrA and HtrB [59]. Here, no correlation between expression of the CssRS targets and *ykrL* was observed, suggesting that *ykrL* expression does not depend on CssRS and is regulated differently from *htpX* in *E. coli*.

In *E. coli*, the membrane located ATP-dependent metalloprotease FtsH is involved in the membrane protein stress response [60]. A similar role of *B. subtilis* FtsH, sharing 47% identity with *E. coli* FtsH, was suggested before [19]. However, *ftsH* was not significantly upregulated in response to overproduction of membrane proteins or to any of the other secretory proteins. Previous results revealing the sporulation control proteins SpoVM and Spo0E as substrates of FtsH [61,62] may therefore be examples of a more specific role of FtsH in *B. subtilis*, rather than a general protein quality control system.

An operon of unknown function, *yvdTSR*, encoding a putative transcriptional regulator and two membrane proteins with homology to small multidrug resistance (SMR) proteins, was also specifically upregulated, but its role in membrane stress is unclear.

Like in case of the other secretory proteins, overproduction of LmrA and XylP led to induction of the class I heat shock protein genes *groES*, *groEL* and class III heat shock protein genes, e.g., *clpE*, *clpC*, which suggests that some fraction of overproduced membrane proteins is targeted by chaperones or proteases for degradation in the cytoplasm before translocation through the Sec machinery and insertion into the membrane. Alternatively, a protein that is incorrectly inserted into the membrane may be subject to Clp-mediated proteolysis, although it is not known whether membrane embedded proteins are accessible to Clp complexes.

### Other extracytoplasmic function (ECF) sigma factors

Next to the SigW response, induced by overproduction of the LmrA, XylP, Usp45 and TEM-1 β-lactamase, upregulation of SigM and SigY RNA polymerase ECF (extracytoplasmic function)-type sigma factors, was observed in some cases (Table 2). SigM has been shown to be involved in a response to salt, low pH, ethanol, heat and oxidative stress and cell wall synthesis inhibiting antibiotics [63,64]. In this study, *sigM* was upregulated under conditions of overproduction of the lipoproteins MntA and YcdH. However, known SigM targets [65] were not upregulated. Expression of SigY and some of the SigY target genes [66] was induced upon XylP and Usp45 overproduction.

## Conclusions

This comparative study revealed differential responses of *B. subtilis* to stress caused by overproduction of secretory proteins with different subcellular localization. New insights in (specificity of) stress responses, in particular at the membrane and cell wall level were obtained. The data reveal possible bottlenecks in the protein production process, which can be targeted in the future development of the improved production strains.

## Methods

### Bacterial strains and growth conditions

Bacterial strains and plasmids used in this study are listed in Table 3. *L. lactis* NZ9000 [67] was used as intermediate cloning hosts for pNZ8901 and pNZ8902 based vectors. *B. subtilis* strains were grown in TY medium [68] at 37°C with vigorous shaking. TY medium was supplemented with kanamycin (5 μg/ml), erythromycin (0.5 μg/ml) or chloramphenicol (5 μg/ml) when needed. *L. lactis* strains were transformed by electroporation as described before [69] using a Bio-Rad gene pulser (Bio-Rad Laboratories, Richmond, California). *B. subtilis* strains were transformed as described before [70].

**Table 3:** List of strains and plasmids used in this study

**Strain/plasmid**	**Description**	**Reference**
*L. lactis*		
NZ9000	MG1363 derivative, *pepN*::*nisRK*	[67]
*B. subtilis*		
NZ8900	168, *amyE*::*spaRK*, KanR, SURE expression system host	[7]
Plasmids		
pNZ8901	SURE expression vector, P*spaSpn*, CmR	[7]
pNZ8902	SURE expression vector, P*spaSpn*, EmR	[7]
pNZ-xynA	pNZ8902 carrying *xynA* of *B. subtilis*	[48]
pNZ-usp45	pNZ8902 carrying *usp45* of *L. lactis* MG1363	[48]
pNZ-mntA	pNZ8902 carrying *mntA* of *B. subtilis*	[48]
pNZ-lmrA	pNZ8902 carrying *lmrA* of *L. lactis* MG1363	[48]
pNZ-nprE	pNZ8901 carrying *nprE* of *B. subtilis*	This work
pNZ-bla	pNZ8902 carrying *bla* of *E. coli,*	This work
pNZ-ycdH	pNZ8902 carrying *ycdH* of *B. subtilis*	This work
pNZ-xylP	pNZ8902 carrying *xylP* of *Lb. pentosus*	This work

### Plasmid and strain construction

Molecular techniques were carried out as described before [71]. All primers used in this study are listed in Table 4. To construct overexpression vectors, the genes *nprE*, *bla*, *ycdH* and *xylP* were amplified using primers nprE-fw and nprE-rv, bla_F and bla_R, ycdH-Fw and ycdH-rv, xylP-fw and xylP-rv, respectively. Template DNA for amplification of *nprE* and *ycdH* was *B. subtilis* chromosomal DNA. The *bla* gene was amplified from pUC18 plasmid DNA [72] and *xylP* from chromosomal DNA of *Lb. pentosus*. The PCR products of *bla* and *xylP* were digested with *Pag*I and *Xba*I and ligated to pNZ8902, which was digested with *Nco*I and *Xba*I, resulting in pNZ-bla and pNZ-xylP. The *nprE* PCR product was digested with *Nco*I and *Xba*I and ligated to pNZ8901 digested with the same enzymes, resulting in pNZ-nprE. The *ycdH* PCR product was digested with *Bst*EII and *Xba*I and ligated to pNZ8902 digested with the same enzymes, yielding pNZ-ycdH. Restriction enzymes were obtained from Fermentas. The sequences of all constructs were confirmed by DNA sequence analysis (ServiceXS, Leiden, The Netherlands).

**Table 4:** Oligonucleotides used in this study

**Oligo name**	**Target**	**Sequence**^**a**^**(5’- 3’)**^***a***^	**Restriction enzyme site**
nprE-fw	*nprE*	CGCAAACCATGGGTTTAGGTAAGAAATTGTCTGTTGC	*Nco*I
nprE-rv	*nprE*	GCGAAATCTAGATTAATGGTGATGGTGATGGTGCAATCCAACAGCATTCCAGGC	*Xba*I
bla_F	*bla*	AAACCCTCATGAGTATTCAACATTTCCGTGTCG	*Pag*I
bla_R	*bla*	ATACGCTCTAGATTAATGGTGATGGTGATGGTGCCAATGCTTAATCAGTG	*Xba*I
ycdH-fw	*ycdH*	GCGAAAGGTGACCGATATGTTTAAAAAATGGAGCGG	*BstE*II
ycdH-rv	*ycdH*	GCGAAATCTAGATTAATGGTGATGGTGATGGTGTGATTTAACCAATAGTGAATCTTTCAGGGC	*Xba*I
xylP-fw	*xylP*	CGCATATCATGAGCGTTAGTATGCAGC	*Pag*I
xylP-rv	*xylP*	GCGAAATCTAGATTAATGGTGATGGTGATGGTGCTTTTGATCGTCAGCAA	*Xba*I

^*a*^ Restriction enzyme sites are underlined.

### DNA microarray analysis

The overexpressed endogenous proteins were XynA, NprE, MntA and YcdH (Table 1). The overexpressed heterologous proteins were TEM-1 β-lactamase from *E. coli*, Usp45 and LmrA (inactive mutant) from *L. lactis* and XylP from *Lb. pentosus* (Table 1). For the overproduction of the proteins, the SURE overexpression system was used [7]. The transcription profile of the control *B. subtilis* strain NZ8900 with empty pNZ8902 vector was compared to an isogenic target strain carrying one of the overexpression constructs: pNZ-xynA, pNZ-bla, pNZ-usp45, pNZ-mntA, pNZ-ycdH, pNZ-lmrA or pNZ-xylP. The target strain containing pNZ-nprE was compared to NZ8900 carrying empty pNZ8901. In total, eight independent microarray experiments were conducted.

Strains harbouring overexpression constructs or the empty vectors pNZ8901 or pNZ8902 were grown overnight in 10 ml TY broth supplemented with appropriate antibiotics and diluted the next day in 50 ml of fresh medium to an OD_600_ of 0.05. At an OD_600_ of 0.6, 0.1% (vol/vol) subtilin-containing supernatant of *B. subtilis* strain ATCC 6633 [73] was added to the growth medium to induce gene expression. After 30 min, 10 OD units of each culture were collected for RNA isolation. All the microarray experiments were performed in three biological replicates essentially as described before [74]. Total RNA was isolated using a High Pure RNA isolation Kit (Roche Applied Science). RNA quantity and quality were tested with a Nano Drop ND-1000 spectrophotometer (NanoDrop Technologies) and an Agilent Bioanalyzer 2100 (Agilent Technologies Netherlands BV), respectively. Amino allyl-modified cDNA was synthesized using the Superscript III Reverse Transcriptase Kit (Invitrogen), purified with the CyScribe GFX purification kit (Amersham Biosciences) and labeled with Cy3- or Cy5-monoreactive dye (Amersham Biosciences). Labeled cDNA was purified with the CyScribe GFX purification kit (Amersham Biosciences). Labeled cDNA concentration and dye incorporation were assessed with a Nano Drop ND-1000 spectrophotometer. The labeled cDNA was hybridized to oligonucleotide microarrays in Ambion Slidehyb #1 buffer (Ambion Europe Ltd) at 48°C for 18–20 hours. Next, microarray slides were washed for 5 min in 2 × SSC (300 mM NaCl, 30 mM sodium citrate) with 0.5% SDS, twice for 5 min in 1 × SSC with 0.25% SDS and for 5 min in 1 × SSC with 0.1% SDS, and dried by centrifugation. The slides were scanned with a GeneTac LS V confocal laser scanner (Genomic Solutions Ltd). ArrayPro 4.5 software (Media Cybernetics Inc., Silver Spring, Md., USA) was used to determine intensities of each spot on the microarrays using a local corners background correction method. Resulting expression levels were processed and normalized using the Lowess method with Micro-Prep [75]. The ln-transformed ratios of the expression levels were subject to a *t*-test using Cyber-T tool [76] resulting in expression ratios and Cyber-T (Bayesian) p values.

### SDS-PAGE and Western blotting

In order to determine the subcellular localization of overproduced proteins XylP, LmrA, MntA, YcdH, XynA, NprE, Usp45 and TEM-1-β-lactamase (Bla) in *B. subtilis*, fractionation experiments were performed essentially as described before [18]. Cells were grown in TY medium. At the OD_600_ of 0.6, protein production was induced by adding 0.1% subtilin containing supernatant of *B. subtilis* strain, ATCC 6633 [7,73] and cultures were further incubated. After two hours, cells were collected by centrifugation (4,000 × g, 4°C, 10 min), resuspended in protoplast buffer (PBS pH 7.2, 20 mM MgCl2, 20% sucrose, 2 mg/ml lysozyme, and Complete protease inhibitors Roche) and incubated 30 minutes at 37°C. Protoplasts were collected by centrifugation (4,000 × g, 4°C, 10 min), resuspended in lysis buffer (50 mM Tris–HCl, pH 8, 2.5 mM EDTA) and disrupted by sonication (Sonics Vibra Cell, Beun De Ronde). Unbroken protoplasts and cellular debris were removed by centrifugation (4,000 × g, 4°C, 10 min). Supernatant was ultracentrifuged (200,000 × g, 4°C, 30 min). The supernatant fraction containing cytosolic proteins was collected and an aliquot was used to prepare SDS-PAGE samples. The pellet was resuspended in solubilization buffer (20 mM Tris–HCl, pH 8.0, 10% glycerol, 50 mM NaCl, 1% Triton-X-100) overnight on a rotor at 4°C. Nonsolubilized membranes were removed by ultracentrifugation (100,000 × g, 4°C, 15 min). Supernatant with solubilized membrane proteins was collected and used for SDS-PAGE sample preparation.

The whole cell extracts were prepared as fallows. 1 OD unit of a culture was collected by centrifugation, resuspended in 150 μl of buffer containing 10 mM Tris–HCl pH 8.1, 20% sucrose, 10 mM EDTA, 50 mM NaCl and 2 mg/ml lysozyme, and incubated at 37°C for 30 min. An equal volume of 2x SDS-PAGE sample buffer (100 mM Tris–HCl pH 6.8, 4% SDS, 1% DTT, 20% glycerol, 0.05% bromophenol blue) was added and the samples were boiled for 5 min.

The extracellular proteins present in the medium were precipitated by adding 200 μl of ice-cold 100% TCA to 1.8 ml of medium and incubation on ice for 1 hour. The mixture was centrifuged and the pellet was then washed with acetone, dried by air and resuspended in 100 μl 1x SDS-PAGE sample buffer. Proteins from the whole cell extracts and the cell and medium fractions were separated on SDS-PAGE gels and transferred to a PVDF membrane. The immunodetection of His-tagged proteins was performed using the Penta-His HRP Conjugate Kit (Qiagen) and ECL detection reagents (Amersham).

## Competing interests

No competing interests are declared.

## Authors' contributions

BCM contributed to the design of the study, has been involved in analysis and interpretation of data and writing the manuscript. HT and OPK conceived the study and have been involved in analysis and interpretation of data and writing the manuscript. BCM, HT, PvdV were involved in acquisition of data. OPK and HT were involved in the design of the study and have given final approval of the version to be published. All authors read and approved the final manuscript.

## Supplementary Material

Additional file 1 **Expression ratios of the *B. subtilis *genes under overproduction of different classes of proteins.** Click here for file
